# Genomic characterization of an Omono River virus isolated from *Culex tritaeniorhynchus* in eastern China

**DOI:** 10.1186/s12985-023-02041-y

**Published:** 2023-04-18

**Authors:** Xiaojuan Lin, Bo Sun, Guifang Liu, Yunjiao Wu, Yao Liu, Feng Ji, Zexin Tao, Aiqiang Xu

**Affiliations:** 1 Shandong Center for Disease Control and Prevention, No. 16992 Jingshi Road, Jinan, 250014 People’s Republic of China; 2 Tianqiao Center for Disease Control and Prevention, No. 90 Wuyingshanzhong Road, Jinan, 250031 People’s Republic of China; 3 School of Public Health, Shandong First Medical University & Shandong Academy of Medical Sciences, Taian, 271000 People’s Republic of China

**Keywords:** Omono River virus, Mosquito, Complete genome, Phylogeny

## Abstract

Omono River virus (OMRV) is a newly reported, unclassified RNA virus in the family *Totiviridae*, which infects mosquitoes and bats. In this study, we report the isolation of an OMRV strain SD76 from *Culex tritaeniorhynchus* captured in Jinan city, China. The cytopathic effect was characterized by cell fusion on C6/36 cell line. Its complete genome was 7611 nucleotides in length, with 71.4–90.4% similarities with other OMRV strains. Phylogenetic analysis based on complete genomes showed all OMRV-like strains can be divided into 3 groups with between-group distances ranging from 0.254 to 0.293. These results revealed that the OMRV isolate had high genetic diversity with those identified previously, and enriched the genetic information of family *Totiviridae*.

The family *Totiviridae* contains a group of non-enveloped, double-stranded RNA viruses. They infect a wide range of hosts including fungi, protozoans, plants, arthropods, fish, and bats, etc. According to the 9th Report of the International Committee on Taxonomy of Viruses (ICTV), *Totiviridae* contains 5 genera including *Giardiavirus*, *Leishmaniavirus*,*Totivirus*, *Trichomonasvirus,* and *Victorivirus* [1]. In recent years, the advent of next generation sequencing technology greatly facilitated our understanding of viral diversities, and many new virus genomes belonging to the *Totiviridae* family have been described, although most of which have not been assigned to appropriate genera [2–4].

Omono River virus (OMRV) is an unclassified member of *Totiviridae* first identified from *Culex* mosquitoes collected in Japan in 2005 [5]. Several other OMRV-like viruses were subsequently identified in China, such as Tianjin totivirus isolated from bat guano in 2007 [6], Shanghai totivirus from *Culex tritaeniorhynchus* in 2007 [7], OMRV strain YJ from *Culex tritaeniorhynchus* in 2012 [8], OMRV strain LZ from *Aedes albopictus* in 2017 [9], and *Culex tritaeniorhynchus* totivirus strain CTV-KL in 2016 [10]. In some of these studies [5–7], the cytopathic effect (CPE) of OMRV-like on C6/36 cell line was characterized by cell fusion. In this study, we reported a new OMRV isolated from a pool of *Culex tritaeniorhynchus* mosquitoes collected in Shandong Province, China.

A total of 1737 mosquitoes were collected in pigpens by using hand-held aspirators in a village located at Jinan city of Shandong Province, China in August 2010. There were 1417 *Culex tritaeniorhynchus* collected accounting for 81.6% of total mosquitoes. Mosquitoes were pooled by 50–100 individuals each, and were homogenized in a mixer mill MM400 (Retsch GmbH, Germany) for 10 min at 20/s with 1.5 ml of MEM (Gibco, USA). After centrifugation and filtration, the supernatants were inoculated on C6/36 and BHK cell lines for virus isolation. Strain SD76 was isolated from *Culex tritaeniorhynchus*. A clear CPE of cell fusion was observed on C6/36 cell line within 24 h after inoculation (Fig. 1). No CPE was observed on BHK cells.

**Fig. 1:**
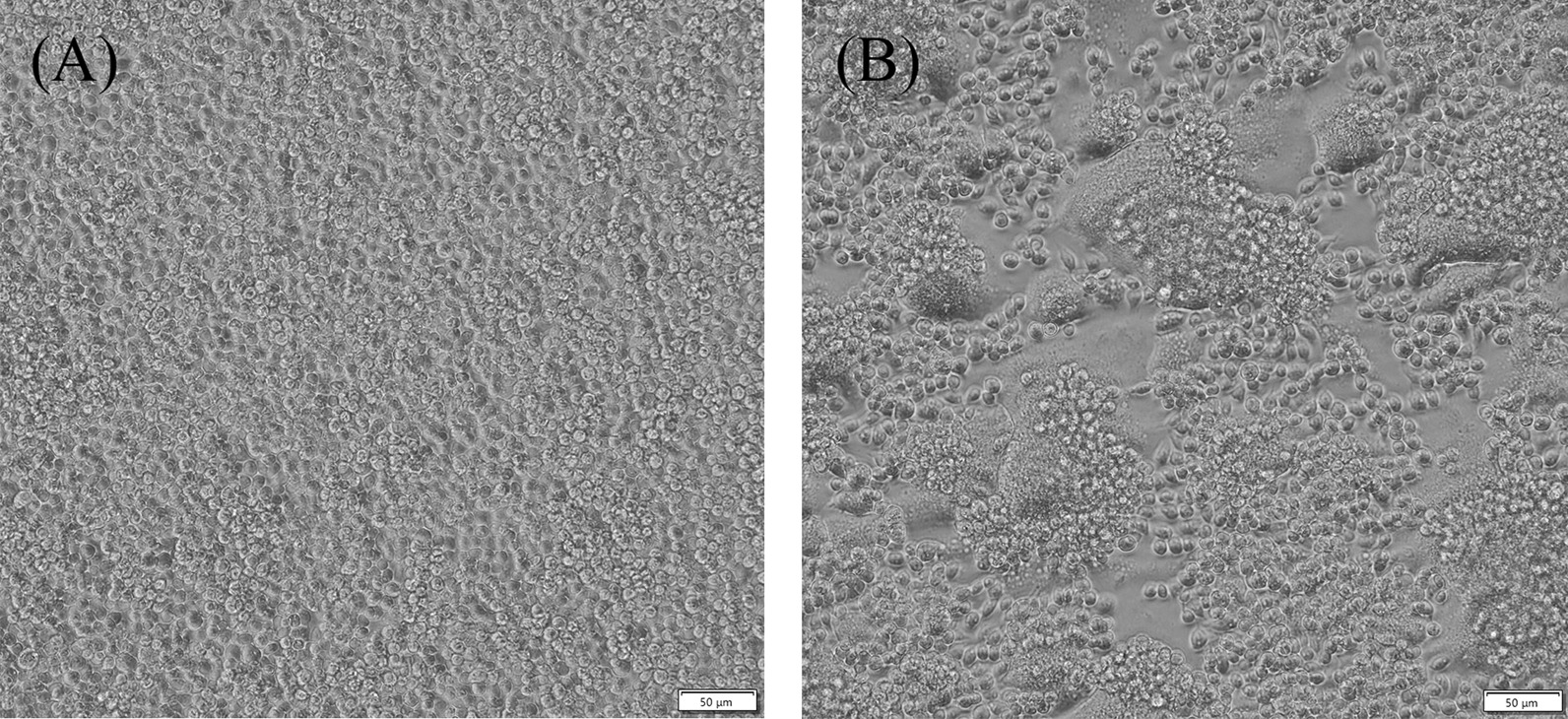
Cytopathic effect caused by OMRV strain SD76 in C6/36 cells. Panel **A** indicates normal C6/36 cells. Panel **B** indicates SD76 infected cells 24 h post-infection

Viral nucleic acid was extracted from the cell culture using MagMAX Pathogen RNA/DNA Kit (Thermo Fisher, Lithuania), and was subjected to BGI (Shenzhen, Guangdong Province, China) for library preparation and sequencing using the DNBSEQ platform. De novo assembly of the clean data was conducted in CLC Genomics Workbench 12.0 (CLC Bio, Qiagen, Hilden, Germany) using the default options. A total of 49,866,666 clean reads (Paired-end, 2 × 100 bp) were generated, among which 16,761,430 (33.6%) reads assembled into a final contig of 7611 nt (Average coverage: 217,573). BLAST analysis showed it belonged to OMRV.

The 5′ end and 3′ end of the viral genome was confirmed using the SMARTer RACE 5′/3′ Kit (Takara Bio, USA) according to the manufacture’s instruction. Gene-specific primers (GSPs) for the 5′-RACE (5′-TGTTGTAGTGTATGATACTCTTCCTT-3′) and 3′-RACE (5′-CTGACACAACTAGCAACACCAACGGC-3′) reactions were designed and used. The products of RACE were Sanger sequenced by using GSP respectively. The results showed that nucleotide sequences in the 5′ and 3′ ends obtained from RACE were 100% identical with those in the NGS contig.

Phylogenetic tree was generated via Mega 11.0 [11] using Neighbor-Joining method based on complete genome sequences. Previous OMRV and OMRV-like genomes available in GenBank were included in the phylogenetic analysis (Fig. 2). All global OMRVs in the tree were divided into 3 clades. SD76 belonged to clade 1 with 6 other sequences from Japan and Guangdong, Yunnan, Shanghai, and Tianjin of China. This clade contained sequences from bat, *Aedes*, *Culex tritaeniorhynchus*, and *Culex pipiens pallens*. The isolation year spanned from 2015 to 2017. Clade 2 contained sequences from China during 2007–2016. They were all isolated from *Culex tritaeniorhynchus*. Clade 3 contained sequences from Japan, China, and Vietnam during 2007–2012. They were isolated from 3 *Culex* species. Clade 1, 2, and 3 had 0.119 ± 0.042, 0.120 ± 0.060, and 0.115 ± 0.099 within mean group distances, respectively. The between group mean distance was 0.254 ± 0.003 (clade 1 vs. clade 2), 0.281 ± 0.003 (clade 1 vs. clade 3), and 0.293 ± 0.003 (clade 2 vs. clade 3), indicating high divergence among these clades.

**Fig. 2:**
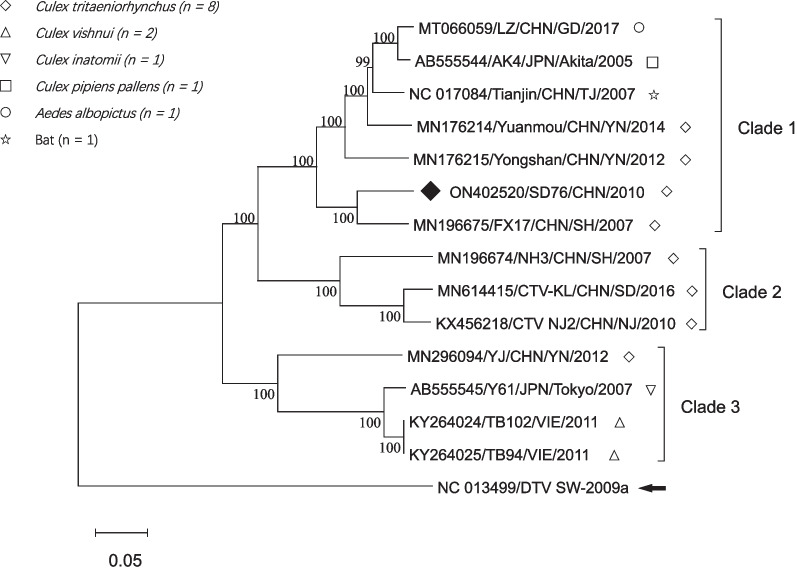
Phylogenetic analyses of OMRV based on complete genomes using Neighbor Joining methods. The percentage of replicate trees in which the associated taxa clustered together in the bootstrap test (1000 replicates) are shown next to the branches. The tree is unrooted. The evolutionary distances were computed using the Kimura 2-parameter method and are in the units of the number of base substitutions per site. All OMRV strains are grouped into 3 clades. ◆ indicates the sequence of this study. The labels on the upper left indicate hosts of OMRVs. The arrow indicates an outgroup (Drosophila melanogaster totivirus SW-2009a)

To understand the phylogenetic relationships with other members of the family *Totiviridae*, a Bayesian tree was derived from the RNA-dependent RNA polymerase (RdRp) amino acid sequences. The multiple sequence alignment was generated using MUSCLE v3.7; the phylogenetic tree was generated using MrBayes v3.2.6 (10,000 generations with sampling every 100 and first 100 generations discarded); and the tree was rendered using TreeDyn v198.3 and refined for presentation using FigTree v1.4.4. All steps were performed at www.phylogeny.fr/ with other default settings [12]. As shown in Fig. 3, global OMRV sequences including strain SD76 formed a cluster distinguishable from all other recognized genera, namely *Totivirus*, *Victorivirus*, *Leishmaniavirus*, *Trichomonasvirus*, and *Giardiavirus*.

**Fig. 3:**
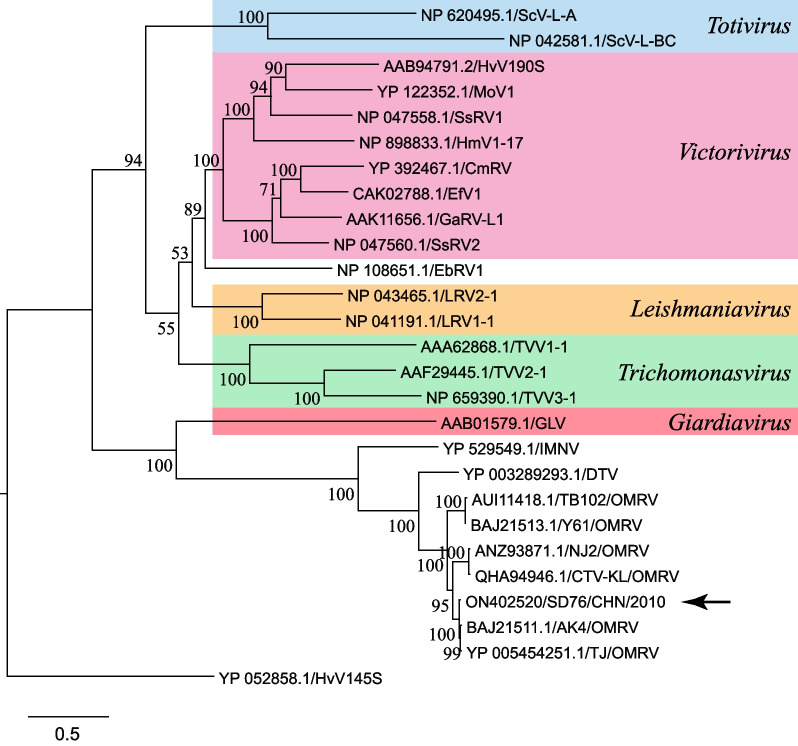
Phylogenetic relationships of OMRV and other 19 viruses in the family *Totiviridae* based on RdRp amino acid sequences*.* The arrow indicates strain SD76 of this study. The tree is rooted on outgroup virus HvV145S (YP_052858) from the family *Chrysoviridae*

Complete genome sequence alignment of OMRVs was performed using the BioEdit 7.0.5.3 software. Strain SD76 had 71.4–90.4% complete genome similarities with previous reported strains, including prototype strains Y61 (71.5%) and AK4 (84.2%). Alignment of amino acid sequences of ORF1 (capsid protein coding region) showed that SD76 had 80.1% similarities with OMRV strain Y61 and 95.4% with strain AK4, and 80.1–98.2% similarities with other strains. Alignment of amino acid sequences of ORF2 region showed that SD76 had 84.6% similarities with OMRV strain Y61 and 98.3% with strain AK4, and 83.0–98.7% similarities with other strains. Sequence MN196675 (FX17/CHN/SH/2007) had the highest nucleotide or amino acid similarities with SD76.

Compared to other genomes of clade 1 and 2, multiple events of nucleotide deletion and insertion were observed in the 4 genomes belonging to clade 3 (stains YJ, Y61, TB94 and TB102). The sites included insertions of 23 nucleotides between 1028 and 1050 positions, 1 nucleotide at 1066 position, and 3 nucleotides between 1933 and 1935 positions, as well as deletions of 9 nucleotides between 1181 and 1182 nucleotide position and 3 nucleotides between 1990 and 1991 nucleotide position. The above positions corresponded to sequence AB555545/Y61/JPN/Tokyo/2007.

Mosquitoes carry a wide variety of viruses. They are important arboviral vectors with the capability to spread pathogenic viruses to humans or animals [13]. The present study reports the isolation of an OMRV from *Culex* mosquitoes. Although most reported OMRV strains were isolated from mosquitoes, a previous study revealed an OMRV-like virus (Tianjin totivirus) was recovered from bat guano [6]. This virus had high complete genome similarity (84.1%) with strain SD76. Since bats play important roles in many ecosystems, it is likely that OMRV might infect more mammalian hosts besides bat. This issue should be addressed in future studies.

It is interesting to note that strain SD76 alongside with Tianjin totivirus, Shanghai totivirus, and two protype OMRVs in Japan can lead to CPE characterized with cell fusion [5–7]. Since *Totiviridae* viruses are non-enveloped, the mechanism of cell fusion induced by OMRVs might be different from other enveloped viruses. Several other OMRV strains such as YJ, LZ, and CTV-KL did not induce cell fusion on C6/36 cells [8–10]. These results suggest cell fusion might not be essential for OMRV proliferation.

In conclusion, an OMRV was recovered from mosquitoes and its complete genome characterization was described. Our data enrich the information of family *Totiviridae*. The virulence, host range, and its potential to infect mammals or even humans need further investigation.

## Data Availability

Condensed anonymized data are available from the corresponding author on reasonable request. Whole-genome nucleotide sequences for the isolate determined in this study have been deposited in the GenBank nucleotide sequence database under the accession number ON402520.

## Abbreviations

OMRV: Omono River virus
GSP: Gene-specific primer
ICTV: International Committee on Taxonomy of Viruses
